# Recent Advances in Pediatric Medulloblastoma

**DOI:** 10.1007/s11910-023-01316-9

**Published:** 2023-11-09

**Authors:** Kasey Jackson, Roger J Packer

**Affiliations:** 1 Brain Tumor Institute, Children’s National Hospital, Washington D C, USA; 2 Division of Hematology and Oncology, Children’s National Hospital, Washington D C, USA; 3 Center for Neuroscience and Behavioral Medicine, Children’s National Hospital, Washington D C, USA

**Keywords:** Pediatric medulloblastoma, Brain tumors, Chemotherapy, Radiation therapy

## Abstract

**Purpose of Review:**

Review recent advances in the understanding of pediatric medulloblastoma including etiology, biology, radiology, and management of pediatric medulloblastoma.

**Recent Findings:**

The classic four subgroups have been reclassified and further subdivided based on new molecular findings. Research is revealing the cell origins of the different subtypes of medulloblastoma. There has been continued personalization of management based on molecular parameters.

**Summary:**

While many advances have been made in the knowledge base of this most common malignant pediatric brain tumor, there has not yet been translation into more effective therapies to prolong survival in all subgroups with the possible exception of children with group 3 disease. Quality of life remains a major challenge for long-term survivors.

## Introduction

Medulloblastomas are embryonal tumors of the posterior fossa. They are the most common malignant brain tumors in children with a median presenting age of 7 years and a male predominance [1]. The most recent WHO classification of central nervous system tumors (WHO CNS 2021 5th edition) has divided medulloblastomas into four molecular subgroups: WNT-activated, SHH-activated and *TP53* wildtype, SHH-activated and *TP53* mutant, and non-WNT/non-SHH (previously groups 3 and 4). These have been further sub-classified into multiple WNT subgroups, four SHH subgroups, and eight non-WNT/non-SHH subgroups based predominantly on methylation testing. Additionally, there are histologic classifications of medulloblastoma that correlate with prognosis and molecular class. These groups include desmoplastic/nodular and medulloblastoma with extensive nodularity (both SHH-activated with good prognoses), large cell and anaplastic with the worst outcomes, and classic (often seen in the WNT group) [2••, 3]. They have now all been grouped as “medulloblastoma histologically defined.” The WHO has suggested that medulloblastomas are classified, whenever possible, by an integrated diagnosis in which the histology, molecular make up, and methylation information are taken into consideration [2••].

## Etiology

A recent analysis found that central nervous system (CNS) tumors have the second highest rate of all childhood neoplasms of harboring pathogenic germline mutations (21/245, 8.6%) and medulloblastomas had the second highest rate within CNS tumors (5/37, 13.5%) [4]. Although most cases of medulloblastoma arise sporadically, they can be associated with multiple genetic predisposition syndromes, especially within the SHH-activated group. Syndromes associated with medulloblastoma include Li-Fraumeni syndrome, Turcot syndrome (*APC*-associated polyposis), some types of Fanconi anemia, and Gorlin syndrome (nevoid basal-cell carcinoma syndrome) [5].

WNT-activated tumors have pathognomonic nuclear staining for beta catenin. This can reflect an activating mutation in *CTNNB1* or an inactivating mutation of the *APC* gene, both of which serve to ramp up WNT signaling. While the latter is a less common manifestation, it should be investigated when a *CTNNB1* mutation is not found. Turcot syndrome can arise when there is a germline mutation in the *APC* gene and results in adenomatous colon polyps in addition to brain tumors like WNT-activated medulloblastoma [3].

The SHH pathway consists of inactivation of suppressor genes *PTCH1* or *SUFU*, activation of the signal transducing molecule SMO, and increased production of the GLI2 transcription factor. This pathway first became associated with medulloblastoma when germline mutations in *PTCH1* were found in Gorlin syndrome. Gorlin syndrome presents with a constellation of skin tumors, craniofacial changes, and an increased incidence of SHH-activated medulloblastomas [3]. Additionally, there is a recently discovered syndrome leading to infant SHH-activated tumors that is caused by germline mutations in *GPR161*. Six such patients have been identified, all demonstrating an initial mutation in *GPR161* (which was found at a frequency of 1:42,000–125,000 in the general population) and then a second hit in the form of 1q loss of heterozygosity [5]. Germline mutations in SUFU can also be seen in those harboring SHH-activated medulloblastoma.

## Biology

Recent studies have elucidated the cells of origin of the different subgroups of medulloblastoma. It is known that medulloblastomas are tumors of the cerebellum which develop from the embryonic rhombic lip. The rhombic lip has two main germinal areas: the ventricular zone which abuts the fourth ventricle and is the site of development for most neurons and glial cells and the external granule layer which surrounds the outside of the cerebellum and produces glutamatergic neurons. The WNT tumors arise from the lower rhombic lip.

By contrast, the SHH pathway acts as a stimulant for growth and division for granule neuron precursor (GNP) cells. Deleting *PTCH1* or inactivating *SMO* in the GNPs leads to medulloblastoma development. It has been shown that similar results occur in GNPs from the cochlear nucleus of the brainstem. Both types of GNPs express *ATOH1*, suggesting that *ATOH1* is a marker of the SHH medulloblastoma progenitor [3, 6••, 7].

Recent studies suggest that the conglomerations of atypical cells that have been known to reside in the cerebellum since the 1940s (persistent rhombic lip—PeRLs) are the precursors to the non-WNT/non-SHH group. In the 1960s, an association was found between these PeRLs and trisomy 17. The PeRLs are a premalignant process and require a second hit to transform into medulloblastoma. A recent study provides evidence that for the formerly labeled group 4 tumors, this second hit is *OTX2* overexpression or *CBFA* complex failure [6••].

## Presentation and Diagnosis

### Clinical Presentation

Posterior fossa tumors (such as medulloblastoma) can result in increased intracranial pressure due to obstruction by the tumor of the flow of CSF. Nonspecific symptoms such as vomiting and headache may occur early on. Headaches occurring just after waking in the morning and with progressive worsening should prompt further workup, including brain imaging. Ataxia is another common presentation of a medulloblastoma, especially gait ataxia due to the frequent midline vermian involvement.

Diagnosis in an infant is often difficult due to lack of localizing symptoms. Open fontanelles and sutures allow for expansion of the head which compensates for the hydrocephalus caused by the growing mass. Presentation can include macrocephaly associated with fussiness and decreased oral intake [7].

Approximately 20–25% of patients have disseminated to other sites in the nervous system, especially the leptomeninges of the spine, at the time of diagnosis. There are usually no specific symptoms referred to metastatic spread.

### Radiology

Medulloblastomas often originate from the top of the fourth ventricle and the inferior medullary velum [8]. The tumors can be quite varied, displaying patchy enhancement, cysts, and sites of necrosis within the tumor [9]. They commonly display hypointense T2 signal and diffusion restriction due to their high cellularity [8, 10]. Some medulloblastomas are non-enhancing or have areas of dissemination that do not enhance. Additionally, small areas of enhancement in the spine can be due to enhanced physiologic nerve roots or increased vascularity rather than tumor spread. CSF findings may help clarify an unclear radiographic picture [9].

The Children’s Oncology Group ACNS9961 study demonstrated the need for quality MRI imaging. Upon retrospective clinical imaging review, average risk medulloblastoma patients (defined as those with no dissemination or post-operative residual disease) who had “good quality” imaging had a 5-year event free survival (EFS) of 83%. This is opposed to patients who did have metastasis at diagnosis that were missed radiographically (5 year EFS 36%), those with significant (greater than 1.5 cm2) residual (5 year EFS 75%), and those who had poor quality imaging studies (5 year EFS 73%) [9].

Goncalves et al. utilized apparent diffusion coefficient (ADC) histogram analysis to provide a diagnosis radiographically for posterior fossa tumors. A total of 59 medulloblastomas were included in the study along with pilocytic astrocytomas (PA), ependymomas, and atypical teratoid rhabdoid tumors (ATRT). There were significant differences in the histogram metrics (*p* < 0.05) between PAs and medulloblastomas and between ependymomas and medulloblastomas, but not between ATRTs and medulloblastomas [11]. Delving even further into radiographic classification, Zhang et al. utilized the Image Biomarker Standardization Initiative (IBSI) on a large sample of pediatric medulloblastoma patients across 12 centers and several countries to radiographically delineate the medulloblastoma subgroups. The paper reported accuracy scores of 95% for WNT classification and area under the receiver operating curve of 98% for defining group 3 versus 4. Gray-level distribution within an image and distribution of voxel intensities (texture and first order statistics) were the most important features in segregating the subgroups [4].

## Management and Outcome

The management of medulloblastoma is multidisciplinary and dependent on the age of the child, risk stratification, and molecular subgroup of the tumor. Conventionally, after surgery, children greater than 3 years of age will proceed to craniospinal radiation with a boost to the tumor site (possibly with concomitant chemotherapy) followed by chemotherapy. Infants have classically been defined as children less than 3 years, but recently as old as 6. They do not receive immediate post-operative radiation therapy but instead often receive intense chemotherapy with stem cell rescue or intrathecal therapy.

### Surgery

It has been shown that those children that undergo the safest maximal resection have better outcomes. Extensive resection often also allows for decompression of hydrocephalus obviating the need for permanent ventriculo-peritoneal shunting. Conventionally, treatment protocols have defined residual disease of greater than 1.5 cm2 (near total resection, NTR) as being a high-risk (HR) feature. This cutoff emanated from the results of the COG-021 trial published in 1999 that was based on CT imaging alone [12]. In 2016, Thompson et al. published an analysis of 787 pediatric medulloblastoma patients and found that there was no statistically significant difference in survival between NTR and gross total resection (GTR) and that extent of resection (EOR) as a prognostic marker was less predictive than other factors. They suggested against grouping children with small residuals into the HR category but did emphasize that there was still merit to performing maximal safe debulking of the tumor [13]. Somewhat in contrast to this is that the recent Children’s Oncology Group study (ACNS0331) analysis of patients, all of whom had central imaging review, found that excess residual/disseminated disease was associated with significantly worse survival outcomes (5-year EFS 81.4% ± 1.9% compared to 56.9% ± 9.1%, *p* = 0.003) [14••].

The need for “total” resection is counterbalanced by the concern that because these tumors can adhere to eloquent structures in the CNS, resection can lead to significant neurologic morbidity. This led to the consensus statement that the definition of > 1.5 cm2 as NTR should be re-evaluated in a clinical trial. In the 20–30% of patients with disseminated tumor, the EOR has not been shown to have independent significance [15].

While the recommendation is to make all efforts to avoid complications from resection, damage to the surrounding cerebellum and brainstem occurs in 5–10% of cases [16–19]. Posterior fossa syndrome (PFS) is a complication that can occur after resection. PFS is also known as cerebellar mutism syndrome because some form of language impairment is usually present. The other associated symptoms can be variable and include ataxia/hypotonia, supranuclear cranial nerve palsies, and emotional lability. It has been reported to occur in 10 to 40% of cases. The only risk factors that have been consistently reproduced are midline location of the tumor and non-SHH type [18]. While the exact cause remains unknown, the current hypothesis is injury to the bilateral dentato-thalamo-cortical pathways [16–18]. Khan et al. analyzed standardized neurologic exams before and after radiation therapy in 175 children diagnosed with medulloblastoma. About one-third of the children developed PFS with two-thirds of those affected having total loss of speech (PFS 1) and one-third having diminished speech (PFS 2). They found that younger age and surgery in a low-volume center (surrogate for surgical experience) increased the risk of PFS, while the SHH subtype had reduced risk (likely secondary to the fact that this type of tumor is usually localized in the lateral hemispheres). Speech returned in all children at a median of about 2 months. Gait had a much more delayed recovery in PFS 2 occurring at a median of 1.5 months versus 0.7 in PFS 1; however, about one-third of the PFS1 group were unable to walk at 12 months. All patients experienced improvement in symptoms; however, none were neurologically normal on exam at a median of 23 months after surgery [17]. A recent publication described a novel Posterior Fossa Syndrome Questionnaire (PFSQ) intended to help improve diagnosis of the syndrome to allow for better consistency in diagnosis and terminology and help promote research [19].

### Staging and Stratification

Patients with medulloblastomas have conventionally been stratified into high or standard/average risk groups to not only prognosticate outcome but also to direct post-surgical adjuvant therapy. Infant medulloblastomas constitute a separate category. In the past, categorization has been based predominately on extent of disease and resection and has not incorporated molecular subtyping. To be classified as standard risk, several criteria must be met: there cannot be metastatic disease (M0, negative cerebrospinal fluid); histology is non-anaplastic; and the amount of post-surgery residual must be less than 1.5 cm2. While there are various protocols available to manage the different strata of medulloblastoma, the main difference in treatment between a standard and high-risk tumor is the amount of craniospinal irradiation (CSI) prescribed (23.4 Gy for standard vs. 36 Gy in high). This is a critical difference, especially for younger children, as higher doses of CSI result in worse cognitive outcomes.

Advances in technology and methylation testing have allowed medulloblastomas to be categorized more exactly. As noted above, there are distinct molecular subgroups. WNT tumors are typically driven by mutations causing activation of exon 3 of *CTNNB1* which leads to the accumulation of beta catenin in the nucleus. This occurs in over 90% of patients [20•]. However, the absence of this accumulation does not disqualify a tumor from the WNT group as there are several other potential drivers. Thus, the standard diagnostic definition is dependent on identification of any two of the following tests: *CTNNB1* immunohistochemistry; *CTNNB1* exon 3 sequencing; gene expression profiling; and DNA methylation profiling. Aside from the common finding of monosomy 6, these tumors maintain a stable genome. These tumors have excellent rates of survival—over 90%—and studies are investigating the safety of de-intensifying treatment. However, Goschzik et al. recently showed that the presence of a *TP53* mutation or *OTX2* gain implies a higher risk of relapse and suggests that such patients should not be considered for de-escalation trials [20•]. The WNT group has also been further divided into alpha and beta classes with alpha occurring in younger ages and more commonly having loss of chromosome 6 and beta seen more in adults. Both sub-groups maintain excellent survival [21].

Cavalli et al. analyzed the other molecular groups of medulloblastoma and subdivided the SHH tumors into four groups, all of which can have desmoplastic histology. The alpha group has a constellation of *TP53* mutations, *MYCN* amplification, metastases, and large cell/anaplastic histology and is associated with poor prognosis. Medulloblastomas associated with germline *TP53* mutation (Li Fraumeni syndrome) are almost always of the SHH group and carry a very poor prognosis [21]. They are separated from *TP53* wild-type SHH tumors in the most recent WHO CNS classification [2]. The beta and gamma groups are often seen in infants, with the beta group having a poor prognosis and the gamma group having a good prognosis with MBEN (medulloblastoma with extensive nodularity) histology. The delta group is associated with *TERT* mutations and is mostly seen in adults [21].

Groups 3 and 4 represent about two-thirds of all pediatric medulloblastoma cases and have now been grouped together as non-WNT/non-SHH [2]. They collectively have a variable prognosis; however, in the setting of *MYC* amplification (seen in the group 3 gamma) the outcomes are poor [20•]. Group 4 tumors commonly display chromatin modifier alterations. Both groups can have whole-chromosome aberrations, isochromosome 17q, and activation of *GF11* or *GF11B* via enhancer hijacking. Northcott et al. analyzed group 3 and 4 tumors together and demonstrated eight subtypes (I–VIII), each with unique cytogenetics and methylation profiles. Others (subgroups I, V, and VII) have demonstrated a common biology between groups 3 and 4 [22, 23••].

The PNET HR + 5 study highlights the utility of molecular subgrouping for stratification. The study concluded that both histology and molecular subtype were significant prognostic factors with large cell/anaplastic tumors having the worst outcomes and SHH and group 3 faring worse than WNT and group 4 (in fact, all group 4 patients, even those with metastases, survived without relapse) [24].

### Postsurgical Management

Through a series of trials, it was determined that the most successful treatment plan for non-infant pediatric medulloblastoma, standard or high risk, was rapid post-operative radiation therapy with both concomitant and subsequent chemotherapy. For infants (the definition of which is in flux, but currently less than 4 years and trending up), radiation therapy has cognitive consequences that must be weighed against the treatment benefits.

Initially, treatment of a standard risk patient was 36 Gy craniospinal irradiation (CSI) with a boost to the posterior fossa up to 54 Gy with goal of preventing leptomeningeal relapse. This regimen had a cure rate of about 60%; the doses of cranial radiation resulted in significant cognitive and endocrine effects on the survivors. The effects on IQ were particularly striking in younger children (3 to 7 years of age). Adding chemotherapy to the treatment plan allowed the lowering of the CSI dose to 23.4 Gy with a 54 Gy boost with similar, if not better, survival outcomes. Unfortunately, lowering the dose of CSI further to 18 Gy did result in poorer outcomes, mostly driven by patients with group 4 tumors. The 5-year EFS for low dose (18 Gy) was 71.4% (95% CI, 62.8 to 80) versus standard dose (23.4 Gy) of 82.9% (95% CI, 75.6 to 90.2) and was inferior to standard dose (hazard ratio 1.67%, 80% upper CI). Further efforts to decrease radiation exposure explored limiting the boost field. The Children’s Oncology Group’s ACNS0331 study showed that limiting the boost field to the tumor bed (versus the whole posterior fossa) did not portend worse outcomes; however, it also did not improve long-term IQ. The 5-year EFS after posterior fossa boost was 80.5% (95% CI, 75.2 to 85.8) versus involved field which was 82.5% (95% CI, 77.2 to 87.8) [14••]. During the time of the study, the paradigm had switched from using photon to proton radiation therapy to decrease “scatter” irradiation. It has been shown that using proton therapy for CSI decreases the radiation to the cochlea and eliminates exposure of the thorax, abdomen, and pelvis [25]. A 2021 study also showed that proton therapy had decreased risk of primary hypothyroidism as compared to photon treatment [26]. Current conventional treatment for non-disseminated patients is now 23.4 Gy CSI with protons up to a 54 Gy boost to the tumor bed with concomitant vincristine followed by cisplatinum based chemotherapy, resulting in a 5-year EFS of about 80–85% [14••].

The Children’s Oncology Group ACNS0332 study evaluated the efficacy of carboplatin as a radiosensitizer in high-risk patients. Results showed that overall, there was no change in survival—5-year EFS with carboplatin was 66.4% (95% CI, 56.4–76.4%) and without, 59.2% (95% CI, 48.8% to 69.6%) with a *p* = 0.11. However, when the data was analyzed by a post-hoc analysis using molecular subgrouping, a significant difference was seen in group 3 patients—5-year EFS with carboplatin was 73.2% (95% CI 56.9% to 89.5%) and without, 53.7% (95% CI, 35.3% to 72.1%) with a *p* = 0.047. However, this was a secondary finding and the study was not powered to differentiate among subclasses. The same study evaluated the efficacy of adding isotretinoin to maintenance therapy in high-risk patients, but this approach was halted early due to futility [27].

A study from France investigated the advantages of neoadjuvant chemotherapy in pediatric medulloblastoma. Though numbers were small, it appeared that giving carboplatin and etoposide prior to surgery increased rates of subsequent GTR without hindering diagnosis or survival and possibly improving neuropsychological outcomes [28]. Further studies are needed on larger cohorts before considering this change in practice.

Infants with medulloblastoma suffer cognitive consequences that are considered too severe to support the use of CSI. Traditionally, patients were classified as “infants” if they were less than 3 years old. Some investigators are recommending that infant protocols should begin at age 4 or older. In lieu of radiation, infants have been treated with a high dose induction chemotherapy regimen followed by even more intense consolidation with stem cell rescue. Alternatively, treating with intrathecal and intravenous methotrexate (MTX) has also been shown to improve survival. The two approaches have been combined and analyzed in the Children’s Oncology Group study ACNS0334. Infants with SHH tumors fared the best (100% 5-year overall survival (OS) both with and without MTX, even with metastases), while those with non-WNT/non-SHH had poorer survival. There is also the possibility of utilizing the high-dose chemotherapy to buy time until the risk/benefit of CSI is more favorable. In the 0334 study, six of 14 survivors required radiation therapy—one SHH and three group 3 patients for residual tumor after chemotherapy and two group 3 patients for disease progression [29].

### Relapse

As with most tumors, cases of relapse or recurrence are often far more difficult to treat than the initial tumor. This is especially the case in older children who have already received multimodal therapy. But regardless of initial therapy, relapses are harder to treat because they can occur with distant spread. This is especially seen in the non-WNT/non-SHH group [30].

Conventional treatment for relapsed medulloblastoma is rarely successful with attempts at re-resection, re-irradiation, and high-dose chemotherapy yielding survival rates less than 10%. Recent studies on relapsed medulloblastoma have revealed that the molecular subgroup does not change; however, certain changes that were possibly actionable in the primary tumor are no longer present at relapse, suggesting that the tumor evolves [30].

Late recurrences (5 or more years after diagnosis) are rare and primarily seen in subgroup VIII of the non-WNT/non-SHH class of tumors [22, 30]. Outside of this, apparent late recurrences are more likely to be treatment induced rather than relapse of the primary tumor. These secondary, often high-grade, glial tumors are resistant to treatment [31]. Re-resection and pathologic diagnosis are important in late or unusual recurrences. Relapse outside of the craniospinal axis is extremely rare.

The use of cell-free tumor DNA (ctDNA) is a promising modality for monitoring medulloblastoma relapse. A recent study by Li et al. showed that “liquid biopsy” using cerebrospinal fluid directly correlated with DNA in the tumor tissue and that tumor-specific DNA markers appeared at times before tumor cells could be detected in the spinal fluid [32]. Additionally, this analysis could be used at diagnosis to help determine the medulloblastoma subgroup (and thus help in risk stratification) prior to surgery. It possibly could also be an aid in detecting tumor recurrence prior to MR detection [33].

### Sequelae in Survivors

Neurocognitive and neuropsychologic sequelae are commonly seen in medulloblastoma survivors of all ages. Sometimes, the damage can be caused simply by the presence of the tumor itself. It has been shown that most infant patients are neurocognitively impaired at the time of diagnosis possibly secondary to prolonged periods of untreated hydrocephalus. Even with the utilization of radiation sparing therapies in infants, it has been documented that most patients will have sequelae. Fay-McClymont evaluated the cognitive outcomes of patients less than 6 years who received high-dose chemotherapy treatment for medulloblastoma (as per CCG 99703) from 1998 to 2011 (nine—about one-third—also received RT: five got focal radiation and four got CSI). Three-fourths of the children studied had low-average to average neuropsychologic (NP) functioning and one-fourth were functioning in less than the tenth percentile in at least one domain [34]. The addition of neurotoxic methotrexate to these regimens my cause even more neurocognitive decline.

Disruptions of the hypothalamic-pituitary axis causing hormonal disturbances are another common side effect of treatment for medulloblastoma. This is often a consequence of exposing the whole brain to radiation. Decreases in all the pituitary hormones are possible, including thyroid stimulating, follicle stimulating, luteinizing, and adrenocorticotropic hormones. However, deficiencies in growth hormone production are most common. The subsequent fall off of linear growth can be compounded by the effects of the radiation on spinal vertebral growth [35]. While the use of a growth hormone in the setting of a tumor with already unregulated growth is controversial, in medulloblastoma it has not been associated with increased rates of relapse [36].

There are also sequelae associated with the various chemotherapeutic agents that are used to treat medulloblastoma. While often limited to periods of treatment, vincristine can be associated with chronic neuropathy. Additionally, cisplatin is known to cause irreversible high-frequency sensorineural hearing loss. Early dose reduction or deletion of drug from further treatment can limit the degree to which hearing loss interferes with frequencies used in daily life. Cyclophosphamide can be associated with secondary malignancies as well as fertility issues.

## Conclusion

Much has been learned in the last few years about medulloblastoma; however, there are still advances to be made in long-term survivorship for all subtypes and quality of life for the survivors. The advent of methylation testing has allowed more refined subclassification and holds the possible promise of more personalized treatment. Strides have been made in reducing treatment toxicity, although survivors continue to have long-term sequelae. Overall, there is still much to be learned about this common posterior fossa tumor.

## Data Availability

Not applicable.
